# Impact of contusion injury on intramuscular *emm*1 group a streptococcus infection and lymphatic spread

**DOI:** 10.1080/21505594.2018.1482180

**Published:** 2018-07-27

**Authors:** L. E. Lamb, M. K. Siggins, C. Scudamore, W. Macdonald, C. E. Turner, N. N. Lynskey, L. K. K. Tan, S. Sriskandan

**Affiliations:** a Section of Infectious Diseases and Immunity, Department of Medicine, Imperial College London, London, UK; b Royal Centre for Defence Medicine, University of Birmingham, Birmingham, UK; c Harwell Science and Innovation Campus, MRC Harwell, Oxfordshire, UK; d Department of Bio-engineering, Royal School of Mines, Imperial College London, London, UK

**Keywords:** Group A streptococcus, contusion, mucoid, lymphatics, capsule, streptococcus pyogenes, hyaluronan, hyaluronic acid, trauma

## Abstract

Invasive group A *Streptococcus* (iGAS) is frequently associated with *emm*1 isolates, with an attendant mortality of around 20%. Cases occasionally arise in previously healthy individuals with a history of upper respiratory tract infection, soft tissue contusion, and no obvious portal of entry. Using a new murine model of contusion, we determined the impact of contusion on iGAS bacterial burden and phenotype.

Calibrated mild blunt contusion did not provide a focus for initiation or seeding of GAS that was detectable following systemic GAS bacteremia, but instead enhanced GAS migration to the local draining lymph node following GAS inoculation at the same time and site of contusion. Increased migration to lymph node was associated with emergence of mucoid bacteria, although was not specific to mucoid bacteria. In one study, mucoid colonies demonstrated a significant increase in capsular hyaluronan that was not linked to a *covRS* or *rocA* mutation, but to a deletion in the promoter of the capsule synthesis locus, *hasABC*, resulting in a strain with increased fitness for lymph node migration.

In summary, in the mild contusion model used, we could not detect seeding of muscle by GAS. Contusion promoted bacterial transit to the local lymph node. The consequences of contusion-associated bacterial lymphatic migration may vary depending on the pathogen and virulence traits selected.

## Introduction

Invasive group A *Streptococcus* (iGAS) infections, such as necrotizing myositis and fasciitis have been historically associated with penetrating trauma [1]. Since the 1990s, invasive *emm*1 (*emm*/M1) infections have been reported in healthy individuals who have a history of blunt, rather than penetrating, trauma [2–4]. A prospective population-based study showed a significant association between such non-penetrating trauma and iGAS necrotizing fasciitis (NF), but not GAS cellulitis [5]. Although traumatic injury appears to accelerate iGAS in some murine models [6,7], it has proved difficult to demonstrate specific seeding of contused soft tissue in the setting of systemic infection, and it remains unclear how infection may arise in the absence of a defined portal of entry.

The main human reservoir for GAS is within the human nasopharynx where GAS employ one or more regulators to undergo alteration of phenotype to survive. One of the most-studied two component GAS regulatory systems is CovRS, which is responsible for regulating 10–15% of the GAS genome, and repressing the expression of virulence genes such as those of the *hasABC* operon that result in expression of hyaluronan capsule [8]. CovRS plays an important role in the systemic dissemination of GAS and spontaneous mutations have been demonstrated in hyper-encapsulated *emm*1 GAS isolates from spleen during experimental *in vivo* infection [9,10].

Few studies have specifically investigated the link between blunt trauma and the development of GAS NF through bacteremic seeding. Using an eccentric contraction model of injury, Hamilton *et al*, 2006, reported GAS seeding of moderately but not mildly injured muscle during bacteremia, potentially linked to GAS binding of skeletal muscle vimentin [6]. To elicit muscle contraction injury, this model required insertion of electrodes through the skin, creating a potential transcutaneous portal of entry, rather than a blunt contusion. Seki *et al*, 2008 showed that mice inoculated intramuscularly with GAS develop a short illness, then recover and remain healthy for about 20 days, but then incur sudden death in the presence of an artificial bruise, elicited by pinching the right hind limb with forceps [7]. The method developed on an earlier model, where a similar delayed deterioration was observed in the absence of trauma [11]. Interpretation of these findings was limited by use of uncalibrated trauma and the possibility of non-specific spread of infection. A key aim of the current project was to develop a calibrated murine model of blunt contusion and undertake a systematic study of both systemic and local (soft tissue) iGAS infection combined with contusion.

## Methods

### Bacteria and bacterial methods

GAS strain H584 [12] and all isogenic derivatives were cultured in Todd-Hewitt broth (THB) (Oxoid) at 37°C with 5% CO_2_ for all experiments. H584 is a contemporary genome-sequenced *emm*1 clinical blood isolate associated with a lethal outbreak of iGAS [12] and was selected as it showed reliable spread to systemic tissues in a mouse model of lower respiratory tract infection. For *in vivo* studies, GAS were cultured overnight, then centrifuged and washed in sterile PBS twice prior to resuspension to the required inoculum density. Capsule hyaluronan from GAS cultured in THB to mid logarithmic growth phase was quantified as described previously [13] using the hyaluronan DuoSet ELISA (R&D). For sequencing analysis of mucoid H584 derivatives arising *in vivo*, streptococcal DNA was extracted then *covRS* and *rocA* were amplified and sequenced using primers described previously [13,14]. *hasABC* promoter primers specifically designed for this work are shown in Supplementary Table 1. Whole genome sequencing of mucoid isolates from lymph node and spleen of one mouse was undertaken using Illumina MiSeq (MRC CSC Core genomics laboratory, Imperial College London). Raw sequence reads have been deposited on the short read archive under the accession numbers: SAMN08125788-SAMN08125792 (five individual colonies from the spleen) and SAMN08125793-SAMN08125797 (five individual colonies from the lymph node). The short read sequences were mapped using SMALT (http://www.sanger.ac.uk/resources/software/smalt/) to the reference *emm*1 genome MGAS5005 (Genbank accession number NC_007297.2) and single nucleotide polymorphisms (SNPs) were identified. De novo sequences were generated using SPAdes [15]. Mapping and SNPs over regulatory regions and the *hasA* promoter were manually checked using Artemis [16].

**Table 1.** Exploratory study, lower respiratory tract GAS infection in presence or absence of trauma: muscle data.

	Number (%) of mice with viable GAS in muscle			
	Injured muscle (left leg)		Control muscle (right leg)	
Time (h) from defined injury to infection	Trauma Group	Control Group	Trauma Group	Control Group
0	0/8 (0%)	0/8 (0%)	0/8 (0%)	0/8 (0%)
24	0/8 (0%)	0/8 (0%)	1/8 (12.5%)	4/8 (50%)
48	1/8 (12.5%)	2/8 (25%)	1/8 (12.5%)	1/8 (12.5%)

n = 8 per group

### Mouse models

Adult female FVB/n mice of 20-30 g, aged 6 to 8 weeks (Charles River UK Ltd) were maintained in individually HEPA filtered cages with sterile bedding. GLP Mini Fun Tunnels (Lillico) were provided for environmental enrichment. Food and water were provided *ad libitum* and the facility was maintained on a 12 hour light-dark cycle. Mice were maintained in cage densities between 3–8 mice per cage depending on individual weights. Mice were anaesthetized briefly with isoflurane (4% induction, 2% maintenance) for intranasal, intravenous infection, and contusion experiments. Following non-terminal experiments, mice were allowed to recover from anaesthesia and return to their cage. Mice were culled if they reached defined humane end points.

#### Contusion model

Under general anaesthesia, a modified weight drop device [17,18] was used to impart a total energy of 156 mJ to the soft tissue of the left hindlimb. Pilot experiments were conducted using two different weights and a height of 13 cm to produce a mild (125 g) or moderate (250 g) inflammatory response in groups of 4 mice. The mild contusion model was used for all subsequent experiments as it did not require the administration of opioid analgesia. Control (non-contusion) animals were anesthetized using isofluorane only.

#### Infection with contusion models

**Figure 1.**
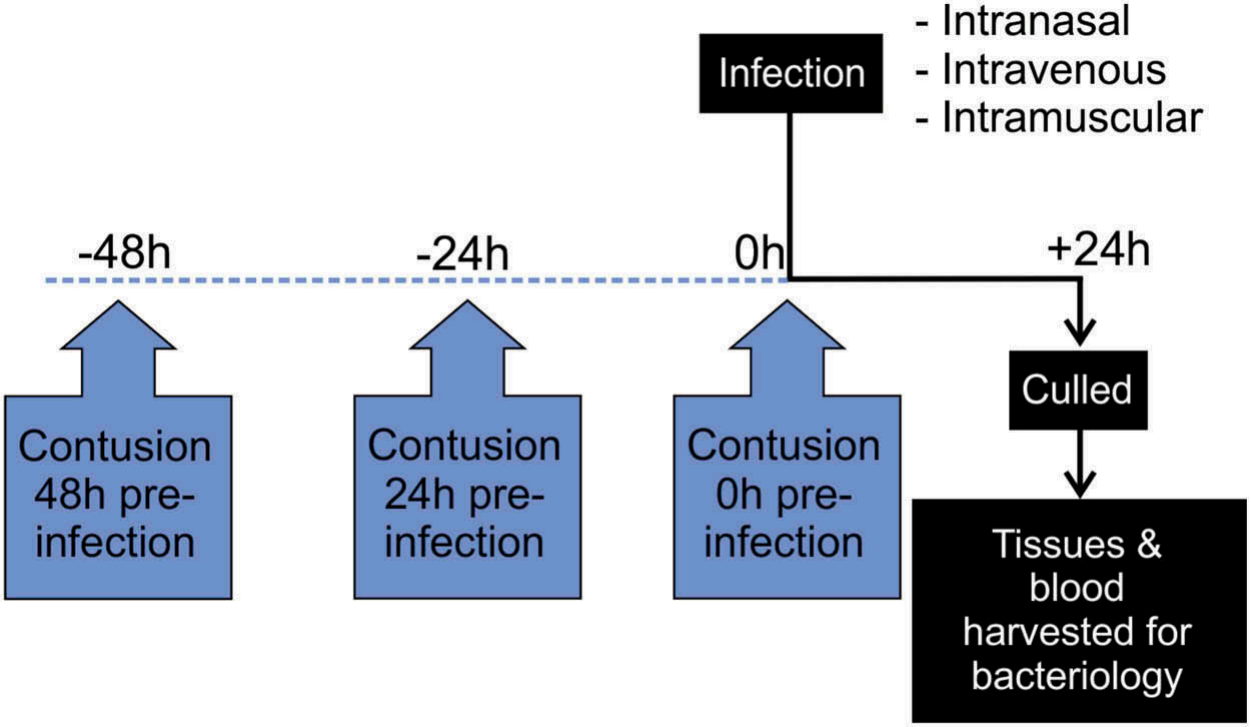
Diagram showing timing of contusion – infection model for exploratory studies. Route of infection and duration of infection initiated at three time points following contusion (0, 24 and 48 hours).

The effect of contusion on progression of *emm*1 iGAS infection was assessed using three routes of infection that were each initiated at 0 h, 24 h, or 48 h after contusion (Figure 1). For exploratory experiments, group sizes of 6 (i.m. and i.v.) or 8 (i.n.) were used. For substantive studies involving i.m. infection, groups were increased to 18 using the same protocols.

#### Lower respiratory tract infection

Following brief isoflurane anaesthesia mice were challenged intranasally with 1 × 10^7^ – 1 × 10^8^ cfu GAS per mouse, administered in a volume of 25 μl that is known to be sufficient to reach the lower respiratory tract [19].

#### Intravenous infection

Following brief isoflurane anaesthesia mice were infected with 1–6 x 10^7^ cfu/mouse in a volume of 50 μl via the lateral tail vein [6] and monitored for 24 h.

#### Local thigh muscle infection

Mice were infected with 1 × 10^8^ cfu in a 50 μl volume by intramuscular injection into the left thigh and monitored for 24 h [20]. This model leads to a systemic infection, weight loss of approximately 10%, and bacteremia at 24 h after onset. Mice were monitored for up to 24 h.

## Bacterial counts

Thigh muscle tissue (right and left), inguinal lymph nodes (right and left), liver, spleen, and lungs (respiratory model only) were harvested from euthanized infected mice. The tissue was weighed and 1x Phosphate buffered saline (PBS) added dependent on the tissue weight. Tissues were dissociated in sterile PBS by fine mincing with dissection scissors; all tissues were subject to homogenization using a sterile plastic rod in a sterile 1.5 mL microfuge tube followed by vortexing. Tissue homogenate was serially diluted in PBS and plated on Columbia blood agar and the number of colony forming units (cfu) per milligram was determined. Blood obtained by cardiac puncture was plated directly on blood agar (5 μl).

## Histopathology

Thigh samples were formalin fixed, decalcified, and paraffin-embedded prior to sectioning and staining with haematoxylin and eosin (H&E) or Gram-staining (when tissue infected). Sections were examined by C.S. (non-blinded) and degree of muscle damage and inflammation scored using a semi quantitative grading scheme[21].

## Statistics

All statistical analyses were performed with GraphPad Prism 6.0. Comparison of two datasets was carried out using an unpaired Mann-Whitney U test or Chi-square test and three or more data sets were analysed by Kruskal-Wallis test followed by Dunn’s multiple comparison tests. A p-value less than or equal to 0.05 was considered significant.

## Ethics

Animal studies were conducted according to protocols approved by the UK Home Office in accordance with the Animals (Scientific Procedures) Act 1986. The protocols were approved by the local Ethical Review Process.

## Results

### Characterization of murine contusion model

**Figure 2.**
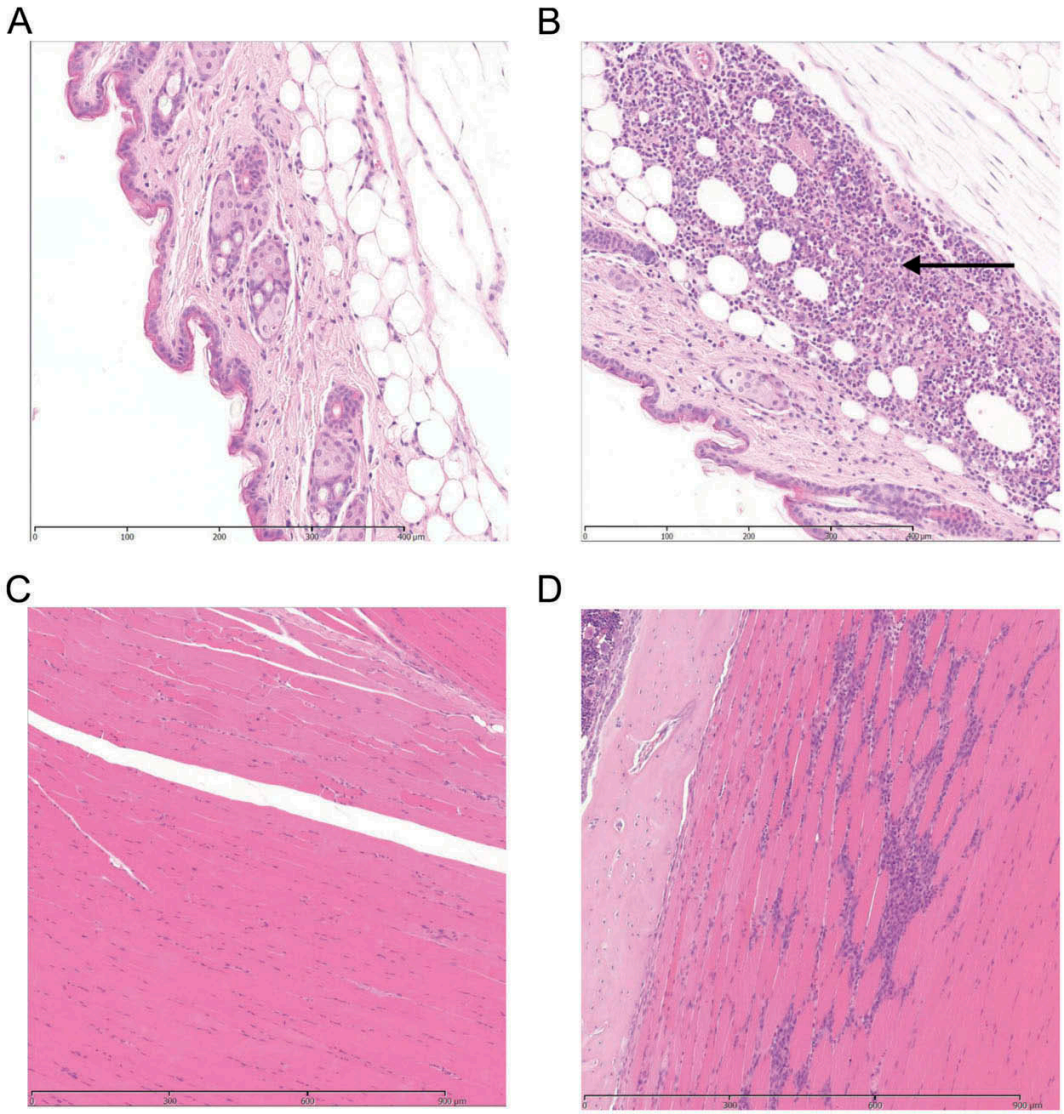
Histological features in soft tissue with or without contusion. Photomicrographs of H&E stained tissue sections 24 and 48 h following contusion. (representative of 12 per group). **A**. Control subcutaneous tissue at 24 h. **B** Focal inflammation in the subcutis of injured leg 24 hours after contusion. Arrow represents the area of injury with an inflammatory response. **C**. Control muscle tissue at 48 h **D** Injured muscle at 48 h after contusion with focal loss of myofibres and replacement by a mixed inflammatory response of neutrophils and macrophages.

To investigate how blunt trauma without a clear portal of entry can lead to iGAS NF infections, we developed a novel murine model of contusion using a modified 125 g weight drop device. This delivered a targeted force of 156 mJ to anaesthetized mice, that resulted in mild inflammation with no bony injury at the site of contusion without any skin break. Histopathological changes in contused tissue comprised mild focal inflammation of the subcutis with infiltration of neutrophils at 24 h, and a mixed inflammatory response of neutrophils and macrophages at 48 h following injury, when compared with controls (Figure 2). A pilot study using a 250 g weight caused moderate myo-degenerative change, inflammatory infiltrates, and occasional fractures without weight loss or clinical signs (Supplementary Figure 1); as such, this weight was not used in subsequent experiments.

### Failure of GAS to localize to injured tissue during bacteremia caused by lower respiratory tract or intravenous infection

It is hypothesized that a low-level bacteraemia, originating from a GAS respiratory infection, could seed sites of soft tissue injury and result in the development of NF. To determine any relation between GAS soft tissue infection and GAS lower respiratory tract infection (LRTI) in the context of antecedent contusion, exploratory studies were conducted in mice intranasally (i.n.) infected with *emm*1 GAS at three different time points (0, 24 and 48 hours) following injury with the mild contusion model (timing as indicated in Figure 1); control groups were infected but not injured. Antecedent contusion did not however enhance specific seeding of GAS to muscle at the site of trauma following LRTI (Table 1). There was also no difference in overall progression of infection at 24 h after onset of LRTI between the injury and control groups, based on the proportion of mice with viable GAS in liver and spleen (Table 2).

**Table 2.** Exploratory study, lower respiratory tract GAS infection in presence or absence of trauma: systemic tissue data.

	Number (%) of mice with viable GAS in tissues							
	Lung		Blood		Spleen		Liver	
Time (h) from defined injury to infection	Trauma	Control	Trauma	Control	Trauma	Control	Trauma	Control
0h	2/8 (25%)	5/8 (62.5%)	1/8 (12.5%)	0/8 (0%)	0/8 (0%)	0/8 (0%)	0/8 (0%)	0/8 (0%)
24h	5/8 (62.5%)	6/8 (75%)	0/8 (0%)	0/8 (0%)	1/8 (12.5%)	3/8 (37.5%)	2/8 (25%)	4/8 (50%)
48h	4/8 (50%)	4/8 (50%)	0/8 (0%)	0/8 (0%)	5/8 (62.5%)	3/8 (37.5%)	4/8 (50%)	2/8 (25%)

n = 8 per group

Although GAS were detectable in spleen and liver 24 h after onset of infection suggesting systemic spread, GAS were not detectable in the blood at the same time point. We therefore undertook exploratory experiments using intravenous injection of GAS to model a more intense or sustained bacteremia. In line with previous work that reported spread of GAS to injured muscle 6 hours following intravenous infection [6], we also detected GAS in injured muscle tissue at 24 h following an intravenous infection. However, importantly, there was no difference between injured muscle tissue in comparison to control muscle tissue taken from the opposite side of individual mice, *nor* was there any difference in bacterial counts between injured muscle tissue from mice subject to contusion and those not subject to contusion, control mice (Table 3). While these data confirmed that GAS can seed muscle tissue from a high cfu bacteremia, the detection of GAS in the contused muscle tissue was a non-specific result of bacteremia, and could not be ascribed to antecedent trauma in this model of contusion. Progression of GAS infection in other tissues was similarly unaffected by contusion (Table 4).

**Table 3.** Exploratory study, intravenous infection in presence and absence of trauma: muscle data.

	Number (%) of mice with viable GAS in muscle			
	Injured muscle (left leg)		Control muscle (right leg)	
Time (h) from defined injury to infection	Trauma Group	Control Group	Trauma Group	Control Group
0*	2/5 (40%)	2/4 (50%)	1/5 (20%)	1/4 (25%)
24	3/6 (50%)	3/6 (50%)	4/6 (67%)	3/6 (50%)
48**	1/4 (25%)	2/5 (40%)	0/4 (0%)	3/5 (60%)

n = 6/group. * 1/6 in the trauma group and 2/6 in the control (uninjured) groups reached humane end points prior to end of study. **2/6 in the trauma group and 1/6 in the control (uninjured) group reached humane end points prior to end of study

**Table 4.** Exploratory study, intravenous infection in presence and absence of trauma: systemic tissue data.

	Number (%) of mice with viable GAS in tissues					
	Blood		Spleen		Liver	
Time (h) from defined injury to infection	Trauma	Control	Trauma	Control	Trauma	Control
0*	4/5 (80%)	4/4 (100%)	4/5 (80%)	(3/4) (75%)	3/5 (60%)	2/4 (50%)
24	3/6 (50%)	4/6 (67%)	5/6 (83%)	5/6 (83%)	4/6 (67%)	4/6 (67%)
48**	4/4 (100%)	5/5 (100%)	4/4 (100%)	5/5 (100%)	2/4 (50%)	5/5 (100%)

n = 6/group* 1/6 in the trauma group and 2/6 in the control (uninjured) groups reached humane end points prior to end of study.

**2/6 in the trauma group and 1/6 in the control (uninjured) group reached humane end points prior to end of study

### GAS clearance from muscle is unaffected by antecedent mild contusion at the same site

**Figure 3.**
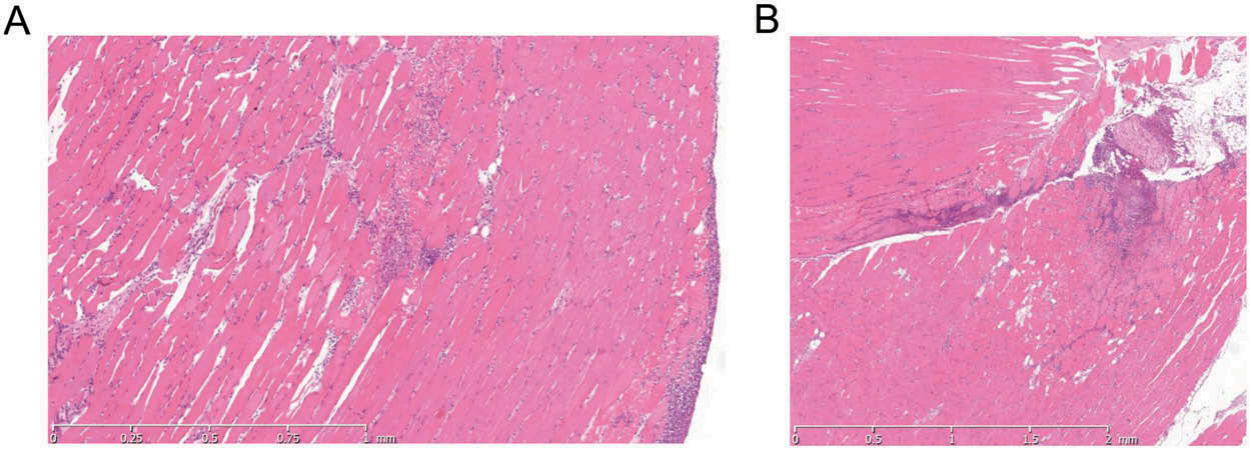
Histological features in soft tissue are similar following infection without or with contusion. H & E stained tissue sections following GAS thigh muscle infection. **A** Photomicrograph of GAS-infected thigh tissue 24 h after infection without contusion. **B** Photomicrograph of GAS-infected thigh tissue 24 h after contusion and GAS infection. Both sections show multifocal mixed inflammatory cell infiltrate with slight myodegeneration and cell lysis. Some haemorrhage is seen in A.

As we were unable to detect specific bacteraemic seeding of contused tissue, we hypothesized that seeding of injured soft tissue may occur via local, unrecognized percutaneous introduction of GAS and could be advanced in some way by antecedent trauma. In a further set of exploratory experiments, mice that received mild muscle contusion were therefore subject to intramuscular infection with 1 × 10^8^ cfu *emm*1 GAS in the same thigh at 0 h, 24 h and 48 h after contusion; mice were then monitored for a further 24 h. Controls were GAS-infected, but did not undergo contusion. The muscle inflammatory response was similar between mice that had combined contusion and infection, and controls that were infected only, with evidence of a mixed cell infiltrate and muscle cell lysis (Figure 3). Antecedent or simultaneous contusion did not affect subsequent GAS burden in injured muscle tissue at any time point examined (Supplementary Figure 2A).

### GAS transit to local draining lymph node is augmented by mild contusion at site of infection

Although GAS clearance from thigh muscle was unaffected by antecedent trauma at any of the time points tested and systemic spread was not augmented by trauma, there was an increase in GAS bacterial counts in the draining ipsilateral inguinal lymph node in those mice subject to simultaneous infection and contusion; this was not seen where infection was initiated after contusion. Indeed, delayed infection appeared to result in protection from lymph node spread (Supplementary Figure 2B, C, D).

**Figure 4.**
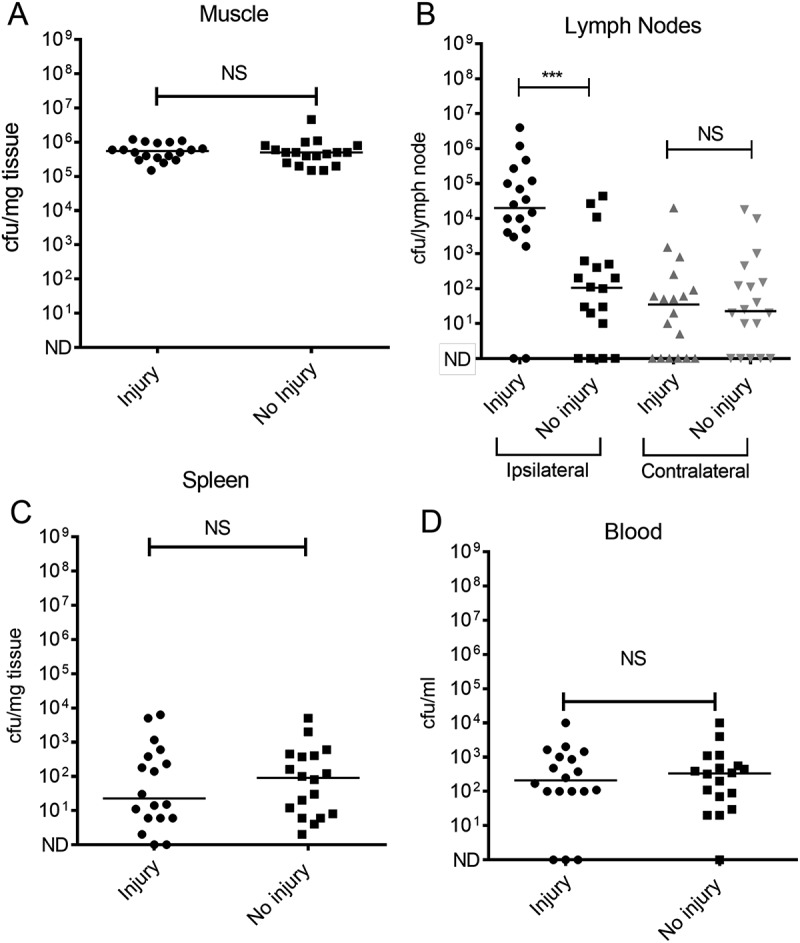
Quantification of GAS in different tissues following simultaneous contusion and local intramuscular GAS infection. Mice were infected i.m. with *emm*1 GAS with or without simultaneous contusion to same limb under anaesthetic. GAS clearance was measured 24h following infection in (**A**) Thigh muscle; (**B**) Draining ipsilateral inguinal lymph node (black) and contralateral inguinal lymph node (grey); (**C**) Spleen (**D**) Blood. n = 18/group for substantive study (includes data from Supplementary Figure 2) Horizontal bars represent the median. Significant difference was observed in bacterial load between contusion group and controls in ipsilateral lymph node in contrast to other tissues (muscle, contralateral lymph node, blood and spleen).NS, non significant difference; ***p < 0.005.

To better understand this phenomenon, we extended the work to undertake a substantive study of lymphatic spread following simultaneous GAS infection and contusion. This demonstrated a significant increase in bacterial counts in the draining ipsilateral inguinal lymph node of mice that were infected at the time of contusion injury, in comparison to control mice that were infected but had not been subject to trauma (Figure 4). Levels of bacteria in the contralateral inguinal lymph node were substantially lower than those found in the ipsilateral draining lymph node. Importantly, no difference in local thigh muscle bacterial burden was observed between injured and control animals, underlining a specific effect of contusion on the interaction of GAS with afferent lymphatics or the ipsilateral inguinal lymph node in these experiments. (Figure 4(a,b)) Although dissemination of GAS to the draining lymph nodes was increased by simultaneous contusion, a difference in systemic spread of GAS to spleen or blood was not detected at 24 h in substantive experiments (Figure 4(c,d)).

### *Emergence of mucoid isolates in draining lymph node associated with a deletion in* hasabc *promoter*

**Figure 5.**
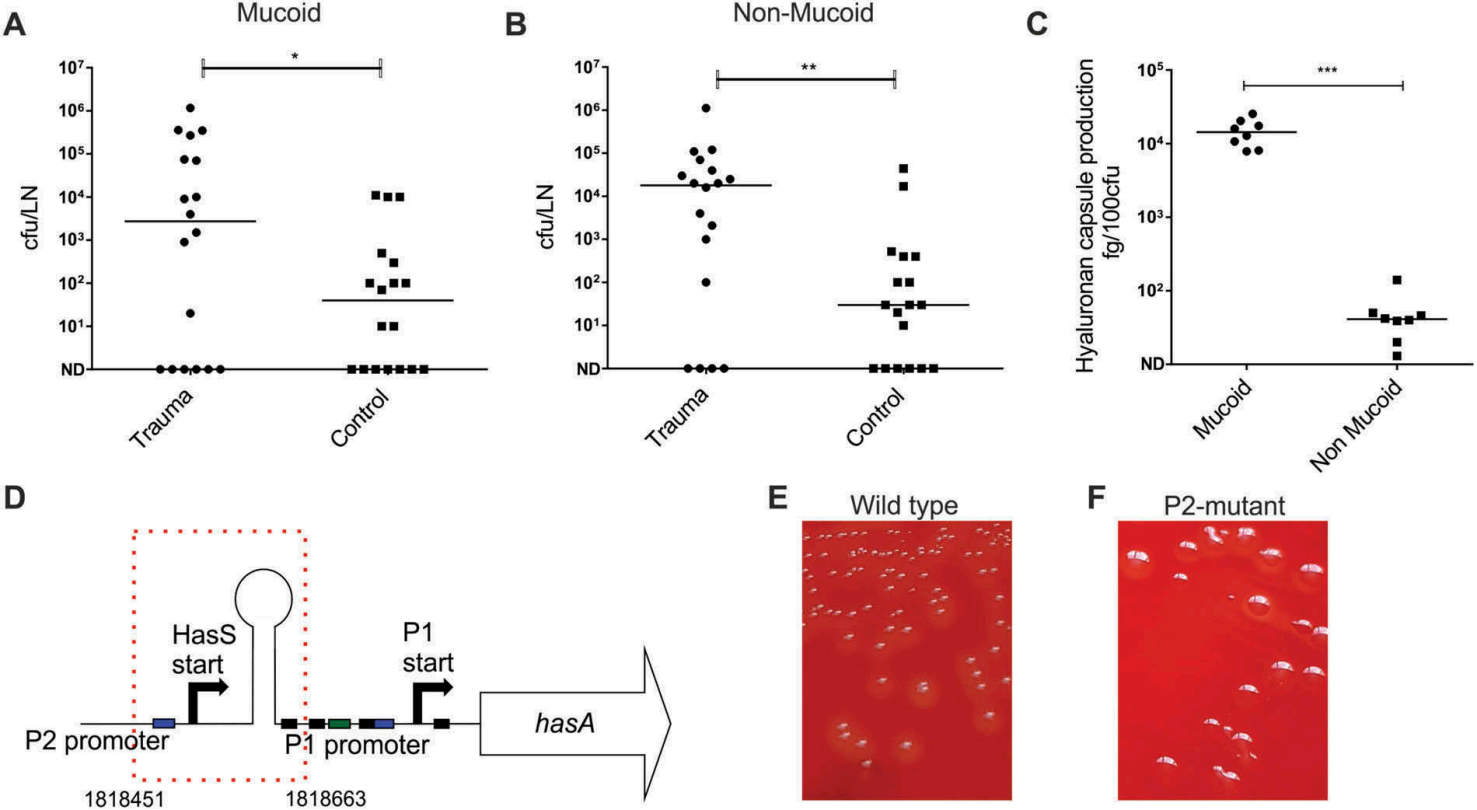
Emergence of *hasABC* promoter deletion variants during GAS infection in draining inguinal lymph node. **A** Number of mucoid colonies and **B**. non-mucoid colonies in draining inguinal lymph nodes of contusion group compared to control mice (*p < 0.01). N = 18 per group (sub-analysis from Figure 4(b)). **C**. Hyaluronan capsule production by mucoid and non-mucoid bacterial colonies isolated from the draining lymph node n = 10 per group (**p < 0.05). **D**. Deletion of 213bp (indicated by red dotted line box) in *hasABC* promoter region identified in all five hyper-encapsulated colony variants from lymph node. Deleted region includes P2 promoter, regulator sRNA *hasS* region, and CovR binding site for the P1 promoter [25,34]. Equivalent position of deletion in completed reference genome MGAS5005 (NC_007297.2) is indicated as 1,818,451-1818663bp. **E**. Colony morphology wild type *emm*1 parent strain and **F**. isogenic hasABC P2 deletion mutant strain

GAS colonies cultured from the ipsilateral inguinal lymph node, blood and spleen of mice infected intramuscularly demonstrated a mucoid phenotype that had not been observed in the inoculum, inter-mixed with colonies that were phenotypically unchanged. When data were analyzed according to colony phenotype, contusion affected spread of both mucoid and non-mucoid isolates to the ipsilateral lymph node (Figure 5(a,b)). Mucoid GAS colony phenotype is usually ascribed to an increase in hyaluronan capsule [13,22] and accordingly, a significant difference in hyaluronan capsule production was observed between mucoid and non-mucoid colonies cultured from lymph node (Figure 5(c)). To determine if hypermucoid strains were accounted for by mutations known to be selected *in vivo*, amplicons of *covRS* and *rocA* [13,23,24], were sequenced from 10 mucoid lymph node colonies, but mutations were not detected. To further determine the basis for hyper-encapsulation, DNA from 5 mucoid colonies cultured from spleen, and 5 mucoid colonies from the draining inguinal lymph node of a single mouse was extracted, subjected to whole genome sequencing, and compared with the parent strain genome sequence. 5/5 mucoid isolates from spleen showed a single nucleotide polymorphism (SNP) in *covR* resulting in a base and amino acid change, ACC (threonine) to CCC (proline) occurring at codon 65 (out of 228). However, in 5/5 mucoid colonies obtained from the ipsilateral lymph node, a 213bp deletion in the promoter of the *hasABC* operon was detected, in a region incorporating the recently described P2 promoter region[25], *hasS* region, and one CovR DNA binding site (Figure 5(d)). Compared to the parental strain, no other SNPs were identified in either the colonies from the spleen or the lymph node. The encapsulated phenotype of the *hasABC* P2 mutant *emm*1 derivative was stable on passage and distinct from the parent strain (Figure 5(e,f)).

### HasABC *P2 promoter variant GAS demonstrates enhanced transit to draining lymph node.*

**Figure 6.**
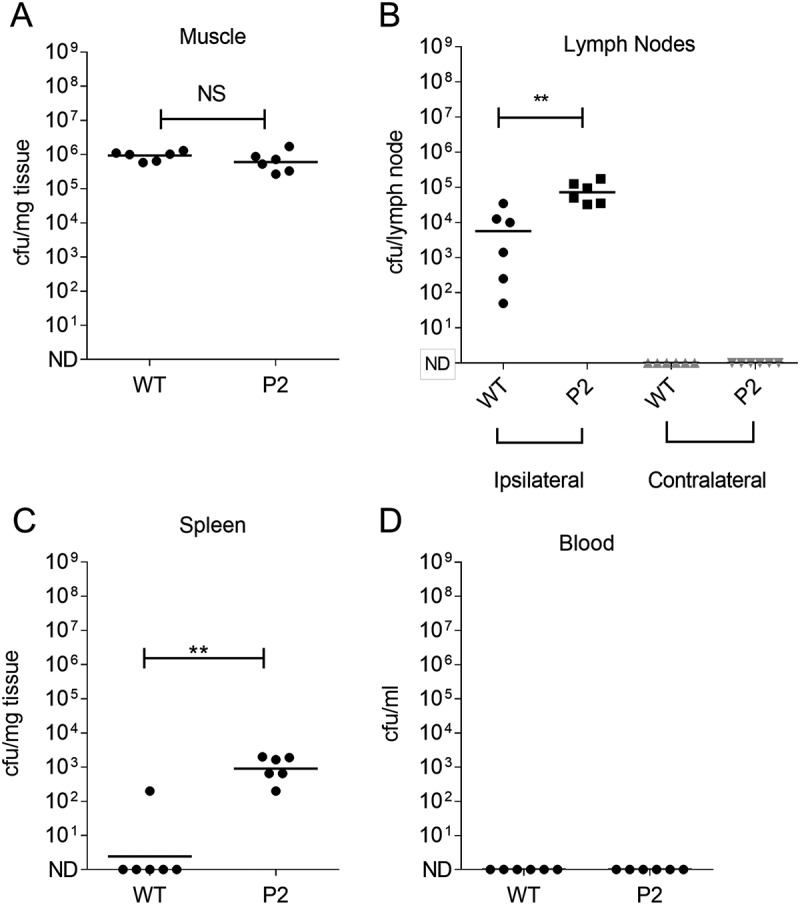
Impact of capsule promoter mutation on GASin ipsilateral lymph node. **A – D** Intramuscular infection with GAS P2 promoter variant (P2) compared with parent *emm*1 GAS strain (WT) (n = 6 per group). GAS colonies were quantified after 3h infection in (**A**) Infected thigh muscle; (**B**) Draining inguinal lymph node or contralateral inguinal lymph node; (**C**) Spleen; (**D**) Blood. P2 mutant showed enhanced bacterial load in the draining lymph node compared to parent *emm*1 GAS strain and also in spleen at this time point despite similar load in thigh muscle (**p < 0.01). Horizontal lines represent the median.

Emergence of the hyper-encapsulated P2 variant was detected in the 5 mucoid isolates cultured from the draining inguinal lymph node, but not spleen, of a single mouse and was detected 24 h after initiation of an intramuscular infection. This raised the possibility that spread to the lymphatic system had resulted in a selective pressure that favoured enhanced capsule expression, or that the P2 variant had enhanced ability to reach the lymph node, either because of reduced clearance in the thigh muscle, or by enhanced affinity for afferent lymphatics, linked to the known hyaluronan interaction with Lymphatic Vascular Endothelial receptor (LYVE)-1[22]. We therefore compared the ability of the P2 variant and the parent *emm*1 GAS strain to reach the ipsilateral inguinal lymph node 3 h after intramuscular infection, in order to focus on afferent lymphatic spread alone, and to reduce the possibility that spread via the systemic (blood) circulation would occur. The data unequivocally demonstrated that, despite an equivalent bacterial burden in the thigh muscle, the P2 mutant had an intrinsically enhanced ability to reach or be retained in the draining inguinal lymph node, and also demonstrated enhanced dissemination to spleen at that time point (Figure 6).

## Discussion

Group A *Streptococcus* (GAS) is widely believed to seed contused tissue after blunt trauma injury [3,6,7]. Using a model of blunt injury with no external skin break, we were unable to demonstrate seeding of an invasive *emm*1 isolate to injured muscle tissue using two models of systemic infection. Furthermore, recent contusion did not affect GAS clearance in muscle at the site of contusion when a local infection was initiated at the same site. Instead, the contusion enhanced transit of GAS bacteria to the locally draining lymph node. Although there was no enhancement of bacterial spread beyond this point, it is conceivable that, if infection had occurred with a more virulent strain, or allowed to progress further, systemic dissemination may have been more rapid. Finally, although shown only once, spread to the locally draining lymph node was associated with emergence of a mucoid hyper-encapsulated phenotype that we determined was due to a mutation in the capsule biosynthesis promoter, and this mutant was shown to have specifically enhanced ability to reach the locally draining lymph node.

Many commentators have highlighted the role played by trauma in the development of invasive GAS infections, particularly necrotizing fasciitis [3,6,7], although the seeding of injured muscle tissue from respiratory tract has not been demonstrated experimentally. Previous studies have reported an effect of antecedent trauma on progression of invasive GAS infection [6,7], although we were unable to confirm specific seeding of contused tissue in our model. Unlike previous work, our contusion model used a reproducible, calibrated method of force, and did not require skin penetration, although did result in muscle inflammation.

We systematically examined data from three routes of infection and three time points using a clinically relevant *emm*1 strain. The contusion applied in the current work was non-severe and we cannot exclude the possibility that a moderate or more substantial contusion would promote bacteremic seeding of contused muscle; nonetheless, increased force resulted in fractures, as reported by other investigators [26–28]. As such, the force applied may be the maximum achievable experimentally. We considered the possibility that the duration of observation was insufficient to detect specific seeding of contused muscle by GAS. It is possible that infection with a less virulent strain might permit more prolonged indolent infection. A pilot study using a 7 day observation period using the *emm*1 strain employed in the current study, failed to demonstrate specific seeding of contused muscle despite heavy bacteremia following respiratory tract infection (Supplementary Table 2). GAS necrotizing myositis and fasciitis are extremely rare events [29] and it could be that localization of bacteria to injured muscle occurs too rarely to be detectable experimentally using group sizes that are practical or ethical in a laboratory setting. Furthermore, there may be additional as yet unrecognized genetic or other risk factors that increase risk of NF in humans.

GAS is known to interact with the lymphatic system as exemplified by well-recognized clinical syndromes such lymphadenitis and lymphangitis [30]. Recently, GAS tropism for draining lymph nodes was reported to be enhanced by a specific interaction between the bacterial hyaluronan capsule and the lymphatic vascular endothelial receptor, LYVE-1 [22]. In the current work, although trauma did not influence the seeding or progression of infection within the muscle, contusion injury did impact on the migration of GAS to the local draining lymph node. We considered the possibility that either lymphatic damage or increased hydrostatic pressure may have enhanced lymphatic spread of bacteria as a direct consequence of the contusion injury. Compression and transient trauma are known to promote lymph fluid movement, and compression techniques are routinely applied in clinical practice to improve tissue viability in cases of lymphedema [31]. The impact of compression on bacteria or large cells, such as cancer cells or leukocytes, is unknown. It seems likely that bacteria such as GAS may be subject to the same mechanical forces that affect lymphatic fluid. We undertook LYVE-1 immunohistochemical staining of control and contused tissues to examine lymphatic channels but could not reliably detect evidence of damage.

The impact of lymphatic tropism on outcome of invasive infection may vary according to the amount of capsule expressed. Whether transit to the lymph node is a protective or dangerous effect may depend on the virulence of the infecting bacterium; for *emm*18 GAS, which are highly encapsulated, reduction of transit to the lymph node was noted to influence development of early bacteremia, whereas this effect was not observed for another GAS genotype [22].

Hyper-encapsulation of GAS *in vivo* was recorded on a number of occasions in the ipsilateral draining lymph node following contusion, although the effect of contusion on GAS migration to lymph node was seen among both mucoid and non-mucoid colonies. To determine the basis for hyper-encapsulation, in one experiment, we undertook genome sequencing of representative isolates from lymph node and spleen from a single mouse. Although isolates from the spleen demonstrated a mutation in *covR*, which might be predicted from many other studies [9,32], the hyper-encapsulated GAS variants in the draining lymph node demonstrated a deletion in the *hasABC* promoter which leads to loss of one of the reported CovR binding sites[25], thereby de-repressing capsule production specifically [25]. The hyper-encapsulated promoter mutant strain (P2) had enhanced fitness with regard to transit to, and/or retention in, the draining lymph node consistent with the reported interaction between hyaluronan and the lymphatic receptor LYVE-1 [22]. The data suggest that altered GAS capsular hyaluronan content affects transit to the draining inguinal lymph node regardless of the mechanism by which capsule content is altered. Although it is possible that the P2 mutation arose *de novo* during infection, perhaps through selective pressure from the innate immune system (as has been reported for *covRS* mutations) or exposure to LYVE-1 receptors, it is equally possible that such mutants are present as rare variants within the injected inoculum; BLAST search did not identify a similar mutation in existing sequences. We tested the possibility that the mutation might emerge again using the same stock of the parent *emm*1 GAS strain, screening colonies from lymph node using primers that amplify the *hasABC* operon, however this did not occur, nor did the mutation arise using an alternate *emm*1 GAS strain. As such, we must assume that selection of the P2 mutation was a rare ‘chance’ event, albeit that the findings were highly informative with regard to the role of increased capsule in lymphatic spread.

In summary, blunt contusion injury in the mouse did not result in detectable seeding of GAS following systemic infection. This may reflect the rarity of invasive GAS infection or the need for additional factors to enhance infection. GAS migration to the local draining lymph node was increased when a mild contusion force was applied at the same time as bacterial inoculation. The findings may be of relevance not only to contusion injury and compartment syndromes, which are recognized to be associated with serious iGAS infections [1,33], but also to the application of compression bandaging in lymphoedema. While this is recognized to enhance lymphatic drainage and overall tissue viability in the lower limb, caution may be required in the context of a more virulent bacterial infection such as GAS until it is known whether such bacterial transit promotes or prevents systemic dissemination.

## Prior presentation of work

This work has been presented in part at the Academy of Medical Sciences Spring Meeting for Clinicians in Training (26 February 2015); the ASM Microbe meeting (July 2016); and ECCMID (April 2015).
