# Supplementary material for: Impact of contusion injury on intramuscular emm1 group a streptococcus infection and lymphatic spread
Source: Virulence. 2018 Jul 27;9(1):1074–84. doi: 10.1080/21505594.2018.1482180 (PMC6068544; doi:10.1080/21505594.2018.1482180)
Supplement: Supplemental Material [file kvir-09-01-1482180-s001.docx]

**Impact of contusion injury on intramuscular *emm*1 Group A streptococcus infection and lymphatic spread**

Lamb LE^1 2^, Siggins MK^1^, Scudamore C^3^, Macdonald W^4^, Turner CE^1 *^, Lynskey NN^1^, Tan LKK^1^, Sriskandan S^1^

^1^Section of Infectious Diseases and Immunity, Department of Medicine, Imperial College London, London, United Kingdom.

^2^ Royal Centre Defence Medicine (Academia and Research, University of Birmingham, B15 2SQ.

^3^ MRC Harwell, Harwell Science and Innovation Campus, Oxfordshire, OX11 0RD UK.

^4^ Department of Bio-engineering, Royal School of Mines, Imperial College London, London, United Kingdom.

- Current address: Department of Molecular Biology and Biotechnology, The University of Sheffield, Sheffield, United Kingdom

**Supplementary Data File**

Supplementary Table 1 Primers used to detect HasABC promoter mutation

Supplementary Table 2 Pilot 7d study of combined contusion and murine lower respiratory tract infection

Supplementary Table 3 Mucoid and non-mucoid colonies in inguinal lymph node in presence and absence of contusion

Supplementary Figure 1 Contusion model using 250g weight: H&E stained thigh tissue

Supplementary Figure 2 Exploratory study: intramuscular GAS infection in presence and absence of local soft tissue trauma: muscle and systemic tissue data.

**Supplementary Table 1 Primers used to detect HasABC promoter mutation**

| ***Has*ABC promoter primers** | Sequence |
| --- | --- |
| **Forward Primer : H584 Has del FWD** | GATGAAGTTGTACTCCCTGAACAA |
| **Reverse Primer : H584 Has del REV** | TGAAAGACAGGGACCTCGAT |

**Supplementary Table 2 Pilot 7d study of combined contusion and murine lower respiratory tract infection**

|  | **Mice with viable GAS bacteria in tissues** | | | |  |
| --- | --- | --- | --- | --- | --- |
|  | **Endpoint reached < 7 days** | | **Survived** | | **Chi square** |
|  | **Trauma** | **No Trauma** | **Trauma** | **No Trauma** | **P value** |
| **Lung** | 7/7 (100%) | 6/6 (100%) | 5/9 (56%) | 1/10 (10%) | p<0.001 |
| **Blood** | 4/7 (57%) | 2/6 (33%) | 0/9 (0%) | 0/10 (0%) | p>0.05 |
| **Spleen** | 4/7 (57%) | 5/6 (83%) | 1/9 (11%) | 0/10 (0%) | p>0.05 |
| **Left Lymph Node** | 3/7 (43%) | 1/6 (17%) | 1/9 (11%) | 0/10 (0%) | p>0.05 |
| **Right Lymph Node** | 3/7 (43%) | 1/6 (17%) | 2/9 (22%) | 1/10 (10%) | p>0.05 |
| **Injured Muscle (L)** | 3/7 (43%) | 1/6 (17%) | 1/9 (11%) | 1/10 (10%) | p>0.05 |
| **Uninjured Muscle (R)** | 4/7 (57%) | 1/6 (17%) | 2/9 (22%) | 1/10 (10%) | P<0.05 |

Table shows numbers of mice (and percentage) with GAS in lung, blood, spleen, lymph node or muscle, as a proportion of total number of mice in group, following intranasal *emm*1 GAS infection with or without simultaneous mild contusion. Data are presented as follows: First two columns provide data for mice reaching humane endpoint prior to 7d, and were obtained by culturing tissues on the date of death (range 1-7 days). The next two columns provide data for remaining mice that survived to 7d; all tissues were cultured at the same time-point and are therefore comparable. The last column provides chi-square values comparing observed and expected number of mice with GAS dissemination (comparing all mice observed). Specific seeding of contused (left sided) muscle was not seen.

**Supplementary Table 3 Mucoid and non-mucoid colonies in inguinal lymph node in presence and absence of contusion**

| Mouse Number | Cfu/ Lymph node | | | |
| --- | --- | --- | --- | --- |
|  | Trauma | | Control | |
|  | Mucoid | Non mucoid | Mucoid | Non Mucoid |
| 1 | 9000 | 16000 | 0 | 30 |
| 2 | 350000 | 120000 | 500 | 0 |
| 3 | 117000 | 1120000 | 11000 | 0 |
| 4 | 10000 | 110000 | 0 | 30 |
| 5 | 4000 | 1000 | 70 | 30 |
| 6 | 1500 | 100 | 10000 | 17000 |
| 7 | 0 | 4000 | 300 | 100 |
| 8 | 900 | 2100 | 10 | 100 |
| 9 | 75000 | 25000 | 100 | 43900 |
| 10 | 270000 | 0 | 100 | 520 |
| 11 | 360000 | 40000 | 10 | 10 |
| 12 | 70000 | 0 | 0 | 0 |
| 13 | 0 | 0 | 0 | 0 |
| 14 | 0 | 20000 | 0 | 400 |
| 15 | 0 | 30000 | 0 | 20 |
| 16 | 20 | 20000 | 10000 | 0 |
| 17 | 0 | 0 | 100 | 400 |
| 18 | 0 | 70000 | 1 | 0 |

Table shows raw data for the CFU per lymph node found in individual mice with and without trauma.


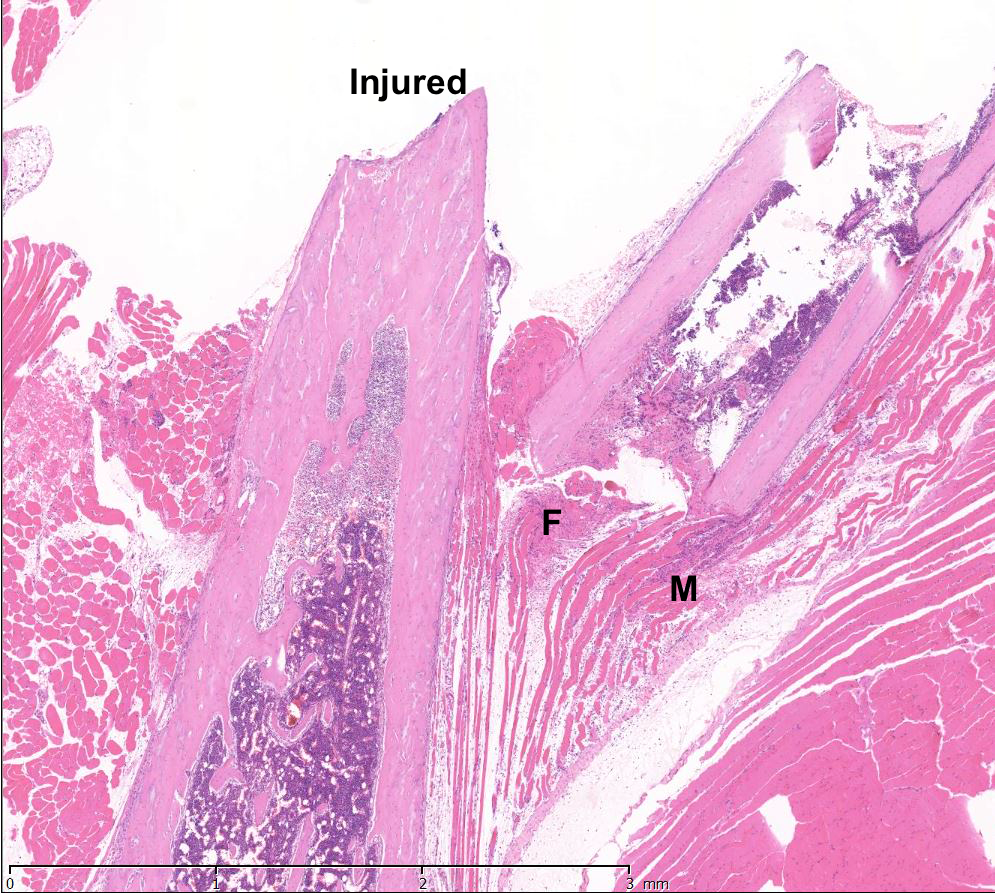


**Supplementary Figure 1 Contusion model using 250g weight: H&E stained thigh tissue** Photomicrograph 24h after moderate contusion using 250g weight showing microscopic evidence of fracture (F) and some muscle necrosis (M). This weight was not used subsequently due to risk of fracture induction.


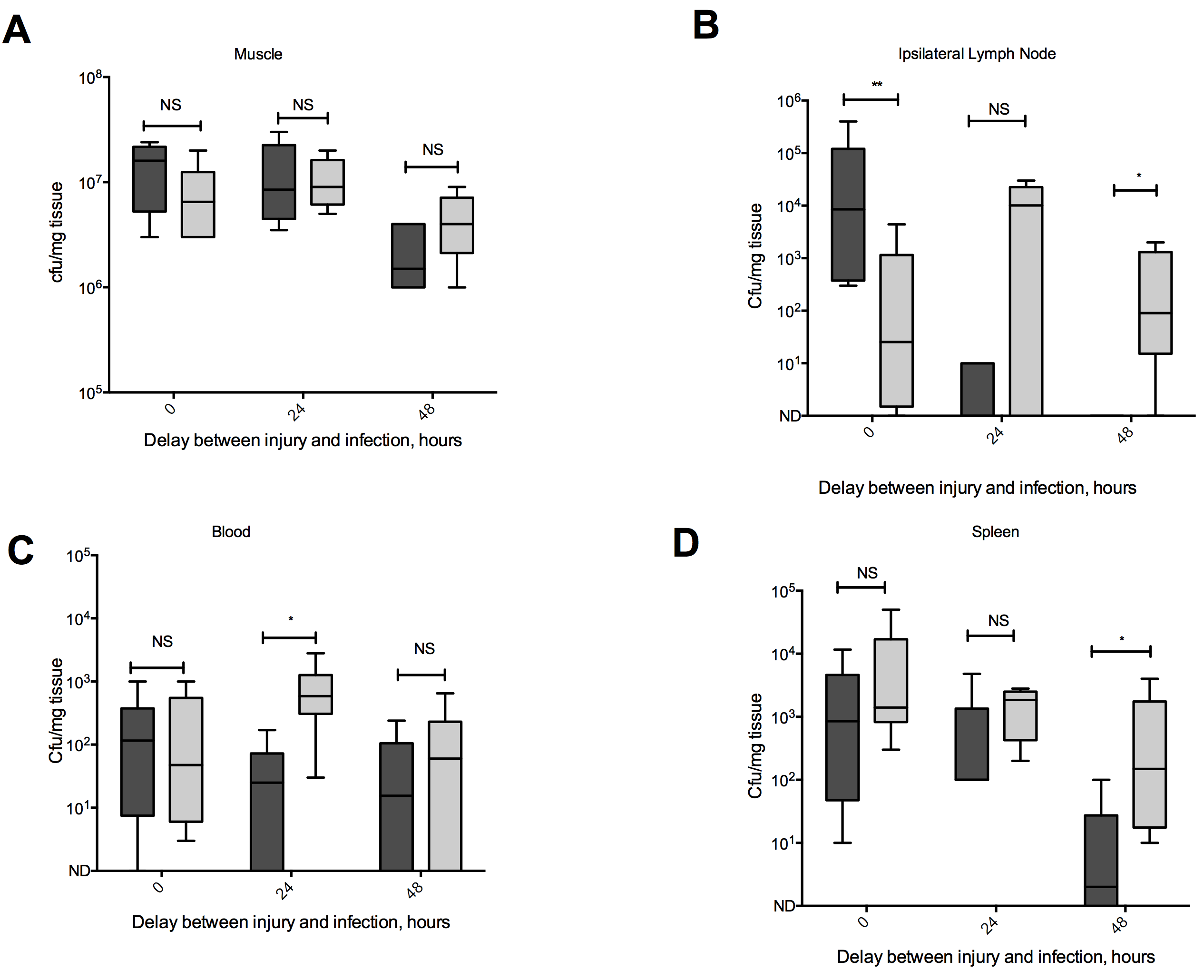


**Supplementary Figure 2 Exploratory study: intramuscular GAS infection in presence and absence of local soft tissue trauma: muscle and systemic tissue data.**

Mice were intramuscularly infected with *emm*1 GAS at 0, 24 and 48 hours following contusion (n=6 per group for exploratory experiment) and observed for 24h (dark grey – trauma, light grey – control). The bacterial load in **A**. muscle tissue, **B.** ipsilateral lymph node, **C**. blood and **D.** spleen was compared to controls. For simultaneous infection with contusion, differences were not seen except that GAS cfu were greater in the draining LN in presence of contusion. With greater delay between contusion and infection, mice appeared partly protected from infection progression. Data contributed to substantive study conducted at 0h following injury (included in Figure 4)
